# Possibilities of using T-cell biophysical biomarkers of ageing

**DOI:** 10.1017/erm.2022.29

**Published:** 2022-09-16

**Authors:** Blanca González-Bermúdez, Aldo Abarca-Ortega, Mónica González-Sánchez, Mónica De la Fuente, Gustavo R. Plaza

**Affiliations:** 1 Center for Biomedical Technology, Universidad Politécnica de Madrid, E-28223 Pozuelo de Alarcón, Spain; 2 Department of Materials Science, E.T.S.I. de Caminos, Canales y Puertos, Universidad Politécnica de Madrid, E-28040 Madrid, Spain; 3 Instituto de Investigación Sanitaria Hospital Clínico San Carlos, IdISSC, Madrid, Spain; 4 Departamento de Ingeniería Mecánica, Universidad de Santiago de Chile, Santiago, Chile; 5 Department of Genetics, Physiology and Microbiology, Facultad de Ciencias Biológicas, Universidad Complutense de Madrid, E-28040 Madrid, Spain

**Keywords:** Ageing, biophysical biomarkers, cell deformability, immunosenescence, T cell

## Abstract

Ageing is interrelated with the development of immunosenescence. This article focuses on one of the cell sets of the adaptive immune system, T cells, and provides a review of the known changes in T cells associated with ageing. Such fundamental changes affect both cell molecular content and internal ordering. However, acquiring a complete description of the changes at these levels would require extensive measurements of parameters and, furthermore, important fine details of the internal ordering that may be difficult to detect. Therefore, an alternative approach for the characterisation of cells consists of the performance of physical measurements of the whole cell, such as deformability measurements or migration measurements: the physical parameters, complementing the commonly used chemical biomarkers, may contribute to a better understanding of the evolution of T-cell states during ageing. Mechanical measurements, among other biophysical measurements, have the advantage of their relative simplicity: one single parameter agglutinates the complex effects of the variety of changes that gradually appear in cells during ageing.

Ageing and the decline of the immune system during lifespan are two interrelated processes, leading to the development of deleterious immune responses. There is abundance of evidence that the survival of an organism is largely dependent on a well-functioning immune system, such as the fact that elderly people are more prone to a variety of diseases, including failure to clear infected cells, senescent cells and malignant transformed cells, as a consequence of age-associated immunological changes (Ref. 1). This phenomenon is broadly referred to as immunosenescence, which is the sum of changes affecting the functional decline of immune parameters observed in all mammals studied so far (Ref. 2). In this article we focus on one of the cell sets of the adaptive immune system, T cells, reviewing biophysical markers of ageing.

## Why to measure biophysical features of T cells

Surface molecules known as clusters of differentiation (CDs) are the most widely used biomarkers of T-cell status. To date, nearly 400 clusters of differentiation have been named using CD nomenclature, ranging from CD1 to CD372 (Ref. 3). The expression of these proteins and other chemical biomarkers are the main markers currently available for T cells. For instance, key markers that have been used to identify senescent T cells are loss of CD28, which enables quick interactions during immunological synapses by binding with ligands present at antigen-presenting cells, such as CD80 of the B7 family, and increased expression of CD57 (Ref. 4). Biochemical changes observed in old adult T cells include an increase in proinflammatory cytokines and decrease in telomere length (Ref. 4). Nevertheless, although molecule properties and molecule content are chief markers of T-cell state, part of the effects produced by T cells and many of the changes they suffer when performing immune tasks may be labelled with the adjective physical, since these effects and changes are associated with geometry, movement, deformation, forces, etc.

Reasonably, the physical features and changes depend on the biochemical characteristics of the cells, that is, molecular characteristics and content, and also on how the molecules are ordered. This internal ordering may be included among the physical properties of the cells. A complete description of the T-cell state would include both molecule content and internal ordering. Examples of internal-ordering features are densities of molecules in different parts of the cell, sizes of organelles and other geometrical aspects, such as the thickness of the cytoskeletal cortex. However, important fine details of the internal ordering may be difficult to detect by such geometrical analyses. Furthermore, these fine details may be associated with specific molecules (e.g. proteins in the cytoskeleton, in focal adhesions or in the nuclear lamina) and a comprehensive characterisation of the cells based on biochemical (quantification of the content of particular molecules) and internal-ordering (size and distribution of organelles and cell structures) parameters would require an extremely large number of parameters. Therefore, an alternative approach for the biochemical–biophysical characterisation of cells consists of performing physical measurements for the whole cell, such as deformability measurements or migration-dynamics measurements: the physical parameters, complementing the commonly used chemical biomarkers, may help to better understanding the evolution of T-cell state during ageing.

**Fig. 1.**
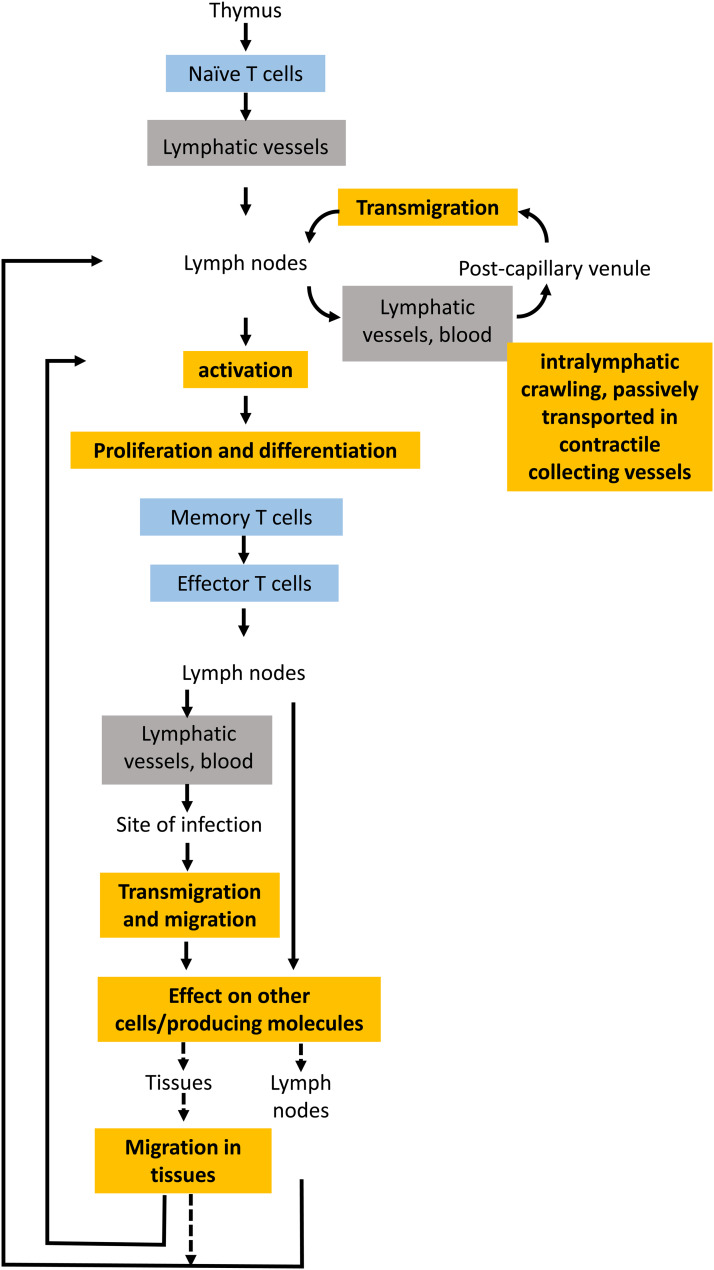
Scheme of the main steps of differentiation, migration and proliferation of T cells. After being selected in the thymus, naive T cells are present in the lymphatic and the circulatory systems. Following activation in a lymphatic node by encountering an antigen presenting cell, naive T cells become effector T cells and some of them migrate and act of the infected tissue. The long-lived memory T cells may be found in tissues (tissue resident memory T cells) and in the lymphatic and circulatory systems (central memory T cells).

The importance of physical features of T cells, and their direct relationship with cell function, is evident when considering their stages of differentiation, migration, proliferation and defensive actions. These routes are represented schematically in Figure 1. The figure shows a brief synthesis of the variety of states of differentiation of T cells. Various studies have proposed that, at least in the case of CD8^+^ T cells, T-cell differentiation proceeds in a progressive, irreversible manner, from naive T cell, to memory stem T cell, central memory T cell, effector memory T cell and finally effector T cell (Refs 5, 6). After recovery from an infection, long-lived memory T cells remain in the body indefinitely, being able to generate a rapid response in the case of a secondary infection (Refs 7, 8). For these cells, the factors that determine tissue residence or lymph-vessel migration are not well known (Ref. 9). Section ‘Direct relationship between biophysical features and T-cell function’ is devoted to the relationships between biophysical properties and T-cell function. Later in this article, the relationships between ageing-related biophysical changes in T cells and their function are analysed in Section ‘Enhancing the biochemical picture: biophysical principles of T-cell ageing’.

### Direct relationship between biophysical features and T-cell function

The term deformability is commonly used referring to the ability of cells to undergo passive deformations. Here the adjective ‘passive’ is used for cell deformation produced by external forces, whereas the adjective ‘active’ is used for the deformation that the cell undergoes when the contribution of cellular molecular motors is required. Non-muscle myosin, acting as a molecular motor, is of chief importance for migrating cells. The deformability of cells is one aspect of their mechanical properties, and it is important in the many occasions in which they undergo both passive and active deformation. Firstly, in lymph vessels, T cells enter and actively migrate within afferent lymphatic capillaries, being this course affected by inflammation-induced expression of cell surface proteins, but are passively transported in contractile collecting lymphatic vessels (Ref. 9) and in blood vessels, needing to reach large deformations (Ref. 10). In addition to the lumen of the vessels, the active transmigration through the wall of high endothelial venules and migration in tissues and lymph nodes depend also on a sufficient deformability. The deformability is important for both CD4^+^ and CD8^+^ T cells since both subsets of cells undergo these same processes. However, the fact that CD4^+^ T cells outnumber CD8^+^ T cells in peripheral blood and in lymph vessels (Ref. 11) suggests that deformability and motility could be particularly important for the first subset. The memory CD8^+^ T cells remain mainly immotile as tissue-resident cells (Ref. 11).

Apart from vessels and healthy tissues, the ability of T cells to migrate in tumours is also of chief importance. T cells penetrate only in the so-called hot tumours, but not in the rest of tumours, known as cold tumours. Hot tumours exhibit an initial immune response that is dampened by upregulation of immune checkpoints or increased numbers of suppressive immune cells (Ref. 12). It is usually assumed that the ability of T cells, particularly effector CD8^+^ T cells, to reach and migrate into tumours is basically determined by signalling molecules expressed by a variety of cells (Refs 12, 13), and therefore the biophysical features of the T cells are, a priori, of minor importance in regard of the ability to migrate into cold tumours.

The geometrical characteristics, in particular the T-cell size, are of importance considering the suitability for transportation in narrow vessels and migration. The diameter of human capillaries is in the range of 5–10 μm (Ref. 10), pore size for endothelial migration of T cells is around 5 μm (Ref. 14) and the average diameter of suspended lymphocytes is approximately 7 μm (Ref. 15). T cells pass through narrow capillaries undergoing large deformations that is assumed to take place at constant volume while increasing their surface, and therefore being the folds of membrane surface reservoirs of critical importance (Ref. 15).

The deformability of cells depends, evidently, on their internal components. In a recent work, the authors proved that the relative size of the nucleus is under certain conditions the most important internal-ordering feature regarding the T-cell deformability (Ref. 16). That work shows that, for memory CD4^+^ T cells from mice of similar age, differences in the content of three cytoskeletal proteins – actin, myosin, vimentin – were of very minor importance compared with differences in the relative size of the nucleus.

### Measurement of biophysical parameters

The deformability of T cells may be quantified by measuring mechanical parameters. Although the cells are complex and nonhomogeneous, the simplest method is to assess the apparent values of mechanical parameters approaching the cell as if it was a homogeneous material (Refs 17, 18). In particular, assuming linear elasticity for sufficiently small deformations, it is possible to assess the apparent Young's elastic modulus, *E* (a constant that measures the stiffness of the material). A more accurate characterisation of the cell consists of evaluating parameters when time-dependency is considered (viscous models). For the large deformations undergone by the cell, for instance when it flows passively in a capillary, it is possible to assess its apparent viscosity, *μ* (Ref. 17). There is a variety of techniques to measure mechanical parameters, including micropipette aspiration, atomic force microscopy, optical tweezers (microscopic beads are optically manipulated to produce deformations and measure forces) and microfluidics-based techniques. The micropipette aspiration technique allows easy evaluating the overall deformability of the cell, by aspirating the cell with a microcapillary (Ref. 18). Atomic force microscopy generally requires the immobilisation of a cell on a transparent substrate and the use of an inverted microscope to position a cantilever with a probing tip, to indent the cell and measure – typically local – mechanical parameters (Ref. 19). In the last few decades the use of microfluidic devices has become important (Ref. 20). In this case, one possibility is to make the fluid with suspended cells to pass through microchannels that display a reduction in their cross section, generating a concentration of the flow and therefore a cell deformation, which can be by contact with the walls of the constrictions or without contact, deforming only by the action of the shear flow.

The cell deformability is key not only when the cells undergo passive deformation but also when they deform actively and produce forces, in particular when they migrate in lymphatic vessels or in tissues. The forces produced by T cells when they interact with other cells may be measured for instance by using microcapillaries (Ref. 18). Basic migration experiments may be performed on two-dimensional substrates. The forces exerted by cells during two-dimensional migration may be measured by techniques such as traction force microscopy (forces exerted by cells on a flexible substrate are quantified by observing the displacements of submicrometric beads embedded in the substrate) (Refs 21, 22, 23) or by the use of micropillars (flexible micrometric pillars are used to assess forces exerted by cells) (Ref. 24). Additionally, kinematic parameters of the migration process may be computed by time-lapse microscopy (a series of images is used to analyse the movement of cells). More realistic experiments, replicating better the physiological environment, may be performed by using microchannels and gels (Ref. 25).

## Approach to the study of age-related changes in T-cells

During ageing, the most evident changes associated with the T-cell population have been described as hallmarks of ageing (see Section ‘Collateral biochemical damage as driver of T-cell ageing’). Most of the changes typically identified in T cells can be labelled as biochemical or functional. In such a description, biophysical changes are not explicitly mentioned, likely because it is assumed that they are an effect of biochemical changes and, in any case, that they are already included in the functional changes. In these three categories (biochemical, functional, mechanical), there are various possible biomarkers to be measured with a variety of available experimental techniques, as explained in the previous section.

The deleterious changes in molecule content predictably affect the properties of the components and organelles and the internal ordering in the cells. Plausibly, biophysical measurements may reflect conveniently these deleterious changes. Mechanical measurements, or other biophysical measurements, have the advantage of a relative simplicity: one single parameter agglutinates the complex effects of the variety of changes introduced in the cells by ageing.

**Fig. 2.**
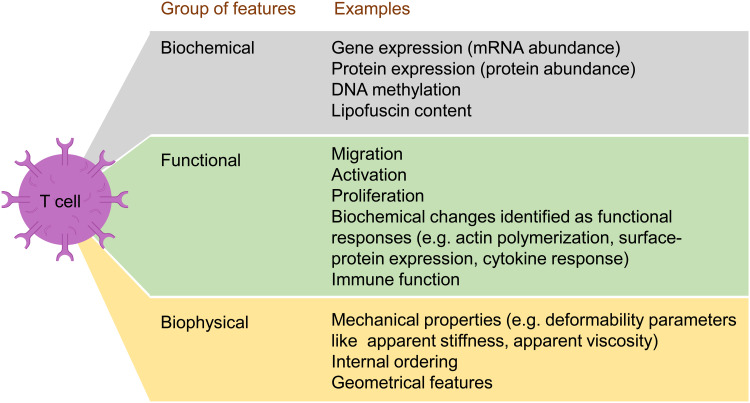
Main examples of biochemical, functional and biophysical features studied for T cells in research works in which the effect of the age of the donor is considered. The figure is based on Table 1.

Figure 2 schematically shows a list of types of features studied in T cells to analyse the effects of ageing. A representative list of studies is shown in Table 1. It is important to remark that a reduced number of longitudinal studies have been performed in comparison with cross-sectional studies. This relative lack of longitudinal studies should prompt new works aiming to describe the evolution of the biophysical properties of the immune system (Ref. 26).

**Table 1.** Representative list of the relatively limited number of works studying T-cell features for various ages of the donors

T-cell source	Biochemical/functional measurements	Biophysical measurements	Main parameters	Ref.
Human healthy young adults and elderly of both sexes; peripheral blood, longitudinal and cross-sectional study	Cell subsets, cytokine response, gene expression		Combined immune-ageing score	27
Mice, healthy young adults and elderly of both sexes; spleen sample; longitudinal study	Cell subsets		Cell subset frequency	28
Human healthy young and mature adults and elderly; peripheral blood; cross-sectional study	Biochemical features, surface-protein expression		Naive/memory ratio, T-cell repertoire, surface-protein expression	29
Human healthy elderly (various age groups) of both sexes; peripheral blood; longitudinal and cross-sectional study	Biochemical features, cell subsets		CD4/CD8 ratio, biochemical biomarkers	30
Human young, old adults and elderly; cytomegalovirus-seropositive; peripheral blood; cross-sectional study	Biochemical features, surface-protein expression		Functional response (surface-protein expression)	31
Human healthy young adults and elderly of both sexes; heparinised venous blood; cross-sectional study	Biochemical features, expression		Proliferation and disappearance rates	32
Human healthy young and old adults; peripheral blood; cross-sectional study	Biochemical features, expression		Functional response (surface-protein expression)	33
Human healthy elderly; peripheral blood; longitudinal study (2-year follow-up)	Biochemical features, expression		Functional response (surface-protein expression)	34
Human donors: Parkinson and control group; venous blood; cross-sectional study	Biochemical features, expression		Proliferation, surface-protein expression	35
Human healthy donors; mononuclear cells and T cells; cross-sectional study		Internal-ordering measurements	F-actin content, functional response (actin polymerisation)	36
Human healthy donors; blood; lymphocytes; cross-sectional study		Mechanical features	Apparent elastic modulus	37
Human healthy adults of both sexes; peripheral blood; cross-sectional study		Mechanical features	Apparent elastic modulus	38
Healthy mice; cervical, axillary and inguinal lymph nodes; T cells; cross-sectional study		Mechanical features	Apparent elastic modulus	39
Human healthy young adults and elderly; peripheral blood; mononuclear cells; cross-sectional study	Functional features		Migration index	40
Human (peripheral blood) and mice (peritoneal lymphocytes); both sexes; healthy; lymphocytes; longitudinal study	Biochemical and functional features		Migration index	41
Healthy female mice: young adult, mature, old, very old; peritoneal T cells; longitudinal study	Protein content, DNA methylation, functional features	Mechanical features, internal ordering, geometry	111 parameters	26

As shown in Table 1, the study of age-related biophysical changes is very limited. The most widely used parameters are biochemical parameters. In this sense, protein expression and genetic changes in T cells have been extensively studied, focusing on changes relevant for the immune function. The reduction of haematopoietic stem cells and the thymic involution result in a reduced number of circulating naive T cells, and increased frequencies of well-differentiated memory CD28− T cells with limited proliferative potential, that is, loss of naive and central memory phenotype with age at the expense of phenotypically distinct CD8^+^ effector T cells, being CD4^+^ T cells more resistant to changes with ageing than CD8 T cells (Ref. 29). The CD4^+^/CD8^+^ ratio decreases with age, and an inverted ratio is associated with short-term mortality (Ref. 34), while reaching an advanced age (over 100 years) was linked to maintaining a high CD4/CD8 ratio (Ref. 30). Also, it has been proposed that a quantification of biological age of the immune system may be performed by using measurements of intracellular signalling responses to cytokines (Ref. 27). These functional responses decrease with age, significantly from the middle age (between 40 and 60 years). Survival was found to be more significantly associated with this quantification than with DNA methylation (Ref. 27).

Migration studies are extensively performed to characterise functionality of T cells (see Table 1). Young individuals show higher T-cell migration ability compared with old individuals (Ref. 26). As a second example of functional feature, the CD4^+^ T and CD8^+^ T cell response to influenza has also been studied comparing young and old individuals, finding increased levels of many proinflammatory cytokines in old individuals, including interleukin (IL)-6 (Ref. 33).

Internal ordering is related firstly to material distribution in the cytoplasm and nucleus. For instance, a cross-sectional study of how ageing affects the content of F-actin in T cells found that the basal F-actin content was significantly higher in lymphocytes from old individuals when compared with young individuals (Ref. 26). In that work, the higher content of F-actin in T cells from old donors was assumed to imply a higher stiffness of the cells. Confirming this assumption, in a recent longitudinal study with mice, it has been shown that the measured stiffness of T cells grows, on average, during ageing (Ref. 26). Regarding mechanical characterisation, it has been studied with special emphasis on the change in stiffness that they may present as an effect of immune responses. The stiffness of T lymphocytes has been studied for resting, activated and apoptotic cells, being significantly stiffer the activated cells and more flexible the apoptotic cells, likely because of structural degradation (Ref. 39). With some similarities to the cell damage produced by ageing, the effects derived from X-ray radiation, a process prior to blood transfusion in many immunocompromised patients, have also been studied in lymphocytes, by measuring mechanical and rheological properties, observing a significant increase in the relaxation characteristic time of those irradiated at 25 Gy compared with native lymphocytes (Ref. 37).

Age-related changes in immune-cell functionality have been studied by quantifying T-cell subset fractions and other relevant features. Such studies established that lymphocyte proliferative responses to mitogens are decreased in old humans and experimental mammals (Refs 42, 43). Cell fraction studies have shown that, apart from thymic involution, ageing results in a biased output of haematopoietic stem cells towards the myeloid lineage at the expense of lymphoid cells (Ref. 44). RNA sequencing, assay for transposase-accessible chromatin sequencing and flow cytometry measurements of peripheral-blood mononuclear cells for men and for women showed a progressive reduction of the fraction of adaptive-immune cells and also epigenetic changes, in both cases being detected earlier and more intensely for men (Ref. 45). Particularly, age-related changes in inflammatory genes/pathways were most significant in men, suggesting an accelerated inflamm-ageing signal (see below) in this sex. Previous studies had established different effects of ageing on CD4^+^ T cell and CD8^+^ T cell subset fractions (Ref. 46).

The interplay between ageing and structural, biophysical and functional properties of innate and adaptive immune cells is still poorly understood, partly because of the difficulty of experimental manipulation of cells without perturbations (e.g. activation), whereas performing in vivo biophysical measurements is challenging by the short life of some cell types such as neutrophils, which has precluded the biomolecular, biophysical and functional analysis of specific populations (Ref. 47). Previous studies with neutrophils reported age-related alterations of F-actin assembly, morphology, deformability and chemotactic migration potential upon stimulation with pro-inflammatory compounds (Ref. 48); these changes have been associated with a decline in neutrophils' ability to extravasate during inflammation (Ref. 47). As red blood cells (RBCs) age, their morphology shifts and their intracellular density increases (Refs 49, 50); these changes have been often attributed to membrane loss, and are associated with RBC stiffening, leading to functional failure in squeezing through inter-endothelial slits (Ref. 51). In macrophages, ageing-impaired F-actin polymerisation was found to reduce alveolar phagocytosis in a mouse model (Ref. 52); these macrophage-dependent changes and the accumulated apoptotic debris promote immune dysfunctions that occur with advancing age and chronic inflammation (Ref. 53).

Overall, the experimental evidence suggest that the biophysical analysis of immune cells may provide a useful framework to better understand the repertoire of changes promoted by ageing, although the primary causes of these changes is still under debate. In the next section, we examine the proposed explanations of the factors that may drive T-cell ageing.

## Explaining T-cell ageing and relationship with biophysical features: theories and models

Several theories purport to explain the ultimate causes of ageing at the molecular, cellular, organ and system levels, and how they give rise to late-life immunosenescence; however, given the multifactorial nature of ageing, involving genetics and environmental factors in a 1:3 estimated ratio, this problem is still open and has led to the emergence of various plausible models (Ref. 54).

Ageing theorists divide the theories that have contributed most significantly to the field into two categories: stochastic (non-adaptive) and non-stochastic (adaptive) theories. Advocates of the stochastic theories sustain that ageing is caused by diverse forms of molecular damage that accumulate with age and lead to the late-life functional decline (Ref. 55). Observations of various types of stochastic age-related cellular damage form the basis of the *damage-based theories of ageing*. On the other hand, the question of whether ageing follows a predetermined sequence of events (a developmental programme) has also been debated for decades (Ref. 56). Although most aspects of *programmatic theories of ageing* do not retain their significance today (Ref. 54), some programmatic features of ageing have been recently subject to revision by Gems and Magalhães because of experimental results from rodents and apes showing an overlap between patterns encoded in the genome and developmental processes (Ref. 56). A paradigmatic shift between the stochastic versus non-stochastic dichotomy was carried by the *hallmarks of ageing* scheme (Ref. 57), focused on how several modes of ageing (primary, secondary or antagonistic and tertiary or integrative causes) might be interrelated, borrowing the idea from a previously published paper on the hallmarks of cancer. The hallmarks of ageing scheme has created a novel perspective on how ageing occurs (it is the most cited article in the field of ageing), although some authors criticise that it explicitly avoids explaining the basic interactions between these hallmarks (Ref. 56).

The hallmarks of ageing were initially drawn from non-vertebrate animal models that lack sophisticated adaptive immunity, so accordingly the ultimate causes of ageing would precede the evolution of T cells, and hence lymphocytes would only play homoeostatic functions unrelated to the ageing process. However, as detailed in the previous section, there is ample experimental evidence in mice that T cells undergo age-related changes of biochemical, biophysical and functional nature. Furthermore, studies of centenarians and supercentenarians suggest that people who reach extreme ages maintain more ‘youthful’ T cell profiles (Refs 58, 59). These lines of evidence suggest that T-cell ageing is linked to longevity and immunosenescence (Ref. 60). This section provides a brief description of theories of various biochemical processes at work in T-cell ageing, and outlines a biophysical model that considers not only biochemical but also biomechanical aspects at work in T-cell ageing, drawing on recent mechanobiological evidence.

### Collateral biochemical damage as driver of T-cell ageing

The problem of revealing order out of the ‘many–many’ relationships between T-cell features and ageing has been addressed by several damage-based theories of ageing. The theories described here put different series of events at the center of T-cell ageing, namely inflammageing, genetic and epigenetic alterations, and cellular senescence.

#### Ageing and the oxidative-inflammatory stress

Ageing is accompanied by events that involve chronic sterile low-grade inflammation, a phenomenon named as inflammageing by Franceschi *et al*. in 2006 (Ref. 61). Inflammageing has its foundation in the observation that organisms are under sustained exposure to various stressor agents over extended time periods. Accordingly, for maintaining life, stressors must be reduced by regulatory mechanisms of the immune systems. Consistent with inflammageing, changes in both the innate and adaptive immune systems occur with age, which are responsible for age-related breakdown of immune regulation, and ultimately contribute to frailty, morbidity and mortality (Ref. 61). Regarding the events of the innate immune system that promote inflammageing, data show that there is an overstimulation of innate immune cells, as reflected by an increase in pro-inflammatory markers with age, such as c-reactive protein, IL-18, tumour necrosis factor-*α* and IL-6, produced by dendritic cells, macrophages and other types of cells (Ref. 62). In concert, inflammation signals induce the recruitment and activation of adaptive immune cells. In this way, T-cell-producing cytokines can initiate further inflammatory responses, for example via Th1 cells, which are able to activate macrophages, both through cell contact and interferon-*γ* secretion (Ref. 63). Furthermore, inflammageing has been related to age-associated changes in gut microbiota that can lead to inflammation, thereby accelerating the rate of ageing (Refs 64, 65).

Considering the phenomenon of inflammageing, it seems reasonable to presume that understanding the nature of ageing requires identifying the causes of inflammageing, considered a key hallmark of the T-cell ageing process (Ref. 60). Based upon the global reduction in the capacity of immune cells to cope with a variety of stressors and inflammageing, the oxidative-inflammageing (oxi-inflamm-ageing) theory proposes links for dysfunctional events leading to ageing (Ref. 66). The oxi-inflamm-ageing theory unifies the ageing theory of oxidation and the age-related changes in immune cells with age.

According to the ageing theory of oxidation, the ageing process is a result of accumulated random molecular damage caused by the high reactivity of free radicals and reactive oxygen species (ROS) produced in cells, as a result of the necessary use of oxygen, which lead to mitochondrial dysfunction in various ways (e.g. free radicals can generate a cellular material rich in lipids and proteins called lipofuscin, which older adults have more of when compared with younger adults). This non-degradable material decreases lysosomal function, which in turns impacts already damaged mitochondria (Ref. 67)). In order to reduce oxygen toxicity, cells count with a variety of antioxidant mechanisms that prevent the formation of ROS or neutralise them once they are produced (e.g. reduced glutathione, capable of neutralising of peroxides (Ref. 67)). However, these systems are insufficient to fully eradicate the damage: when the ROS level exceeds the antioxidant level, an oxidative stress state appears resulting in cell dysfunction.

This oxi-inflamm-ageing theory developed by De la Fuente *et al*. propose that ageing is linked with the excess of chronic oxidative stress, which especially affects cells of the homoeostatic systems (nervous, endocrine, immune) (Ref. 68). As a consequence, these regulatory systems lose their ability to preserve their redox state, which leads to a loss of function compromising homoeostasis (Ref. 66). In this theory, a key role is attributed to the immune system, as the deregulation of immune cell responses, increased by oxidative stress, leads to an increase in the production of proinflammatory cytokines. This increase produces a low-grade chronic inflammatory state that contributes to the generation of ROS, thus producing a vicious cycle of oxidation–inflammation–oxidation (Ref. 67). Oxidative and inflammatory compounds are continuously required by the immune system cells to perform defense functions. If the immune system is dysregulated, it can activate nuclear factor-*κ*B (NF-*κ*B), which in turn has been related to many chronic inflammatory disease states: when a certain level of NF-*κ*B activation is reached, the expression of genes that programme the production of inflammatory compounds is activated, contributing to the aforementioned cyclical process. In this way, both oxidative stress and inflammatory stress, by impairing physiological homoeostasis, would cause oxi-inflamm-ageing.

The oxi-inflamm-ageing theory accommodates previous ageing theories developed since the 1950s, such as *the free radical theory of ageing* (Ref. 69), which offered a mechanistic cause of ageing, wherein ROS species generated as a consequence of metabolism randomly damage cells, with this damage gradually accumulating and resulting in senescence, or *the oxidative mitochondrial theory of ageing* (Ref. 70), which states that the mitochondria are probably the first target of oxidation as oxygen is mainly used in respiration for metabolic processes, thus leading to vulnerability of the mitochondrial genome in differentiated postmitotic cells. Although this theory provides an ordered perspective of mechanistic contributors to immunosenescence, one fundamental question that remains to be addressed is whether the random accumulation of oxidative molecular damage over time is sufficient to cause ageing. Following oxidative damage, the theory proposes that accumulated damage is the main driver of cellular ageing. However, it is unclear why somatic cells cannot remove or repair this particular damage, or decrease its generation by evolving more efficient maintenance mechanisms. Yet interestingly, the oxi-inflamm-ageing model has given guidance on how to efficiently search for biomarkers of age-related diseases and interventions (Refs 71, 72). A number of studies have been set up to focus specifically on robust biomarkers based on inflammageing markers of T cells, as immune cells favour the production of pro-inflammatory over anti-inflammatory cytokines, contributing to the accumulation of cellular and molecular damage in ageing tissues. For further reading, Dufour and Larsson provide an overview on the global measures in T cells of oxidative damage and how oxidants and antioxidants affect longevity (Ref. 73).

#### Ageing and genetic and epigenetic alterations

Another major view in the understanding of T-cell ageing is based on the findings supporting that, over time, cells accumulate changes in the genome and epigenome, ultimately contributing to T-cell ageing. In the past decade, evidence has been collected from the genome analysis of aged T cells. Alteration of several gene networks and pathways that are associated with aged T cells have now been identified in humans and mice, including T-cell receptor (TCR) and activation-related molecules, alteration of chemokine/chemokine receptor expression, gain of natural killer cell receptors and function (Ref. 74). Whether these identified alterations of gene expressions occur in all cells or in subsets of defined T-cell populations remains to be determined. In addition, it is not really clear whether the wild-type genotype affects ageing via (i) accumulated molecular damage, (ii) antagonistic pleiotropy (including trade-offs) or (iii) programmatic mechanisms including futile programme run-on and costly programmes (Ref. 56).

Changes in the epigenome also have a large influence on T-cell ageing, through age-dependent changes in the level of histone protein concentrations and DNA methylation and histone modifications that alter chromatin structure and accessibility. Recently, novel techniques enabled the performance of genome-wide gene-specific epigenetic studies in infrequent cell populations such as T-cell subsets. In particular, chromatin accessibility mapping has been informative to define the epigenetic state of naive T cells versus effector or memory T cells and follow the chromatin changes that occur in antigen-specific T cells when they are activated in a viral infection and differentiate into various effector T cells and memory T cells (Ref. 75). These studies have led to the recognition that T cells undergo large changes in chromatin structure with more than 20% of accessible sites either opening or closing (Ref. 76). For further reading, Chen *et al*. review the gene networks and signalling pathways that are altered with ageing in T cells (Ref. 74), whereas Goronzy *et al*. review how studies of the epigenetic landscape in human T cells are beginning to be informative to understand the mechanisms that drive T-cell ageing (Ref. 75). Certainly, more studies are needed to dissect the primary causes of the impaired genetic and epigenetic expression of age-related genes and their consequences. In fact, rather than being a driving force of ageing, genetic and epigenetic alterations could be correlates of prior events causing T-cell ageing: epigenetic and genetic changes might represent the effects of T-cell ageing rather than the primary causes. Because age-related epigenetic changes in T cells provide molecular correlates of chronological age in human and vertebrates, they are useful for evaluating rates of ageing and interventions focused on healthy ageing (Ref. 75).

#### Ageing and T-cell senescence

Accumulation of senescent cells is considered to be an additional driver of age-related phenotypes in many cells. Cellular senescence can be defined as the stable exit from the cell cycle in response to various stimuli. Senescent cells are metabolically active and participate in diverse effector programmes depending on the type of cell. Replicative senescence is thought to be beneficial for tissue homoeostasis but, if senescent cells persist in tissues, they can be detrimental to the tissue microenvironment, participating in pathological conditions. Increasing evidence indicates that some cell types gradually acquire a secretory phenotype called senescence-associated phenotype (SASP), which is a highly heterogenous feature of senescence (Ref. 77). SASP contains a variety of factors, including inflammatory proteins, cytokines, chemokines, growth factors, and matrix-remodelling enzymes which negatively influence tissue homoeostasis. The most general biomarker of senescence is senescence-associated beta-galactosidase; other markers such as lipofuscin are often used when the studies cannot be limited to fresh tissue samples (Refs 78, 79). Recent works on the search for specific markers associated with T-cell senescence propose that they can be valuable biomarker candidates for the chronic inflammatory phenotype (Ref. 80).

To define such biomarkers, it is necessary to distinguish between exhausted and senescent T cells. Data show that T cells become ‘senescent’ cells when (i) they express CD57 and killer-cell lectin-like receptor subfamily G member 1 (KLRG-1) but show a downregulation of CD27 and CD28, (ii) are resistant to apoptosis increasing the accumulation of memory T cells and (iii) adopt a pro-inflammatory profile (Ref. 81). On the other hand, ‘exhausted’ T cells (I) express various markers associated with programmed cell death, lymphocyte activation and cytotoxic genes (PD-1, LAG-3, TIM-3, CTLA-4) and (II) are unable to proliferate and secrete molecules upon stimulation. Considering T-cell replicative senescence, it has been suggested that rejuvenation of such T cells would provide beneficial age-related immune responses, such as an improved anti-influenza vaccine response (Ref. 82). Nonetheless, the senescent phenotype in T cells is highly heterogenous, and the SASP profile remains to be well defined in T cells (Ref. 83). Indeed, the premise that T-cell ageing is partially governed by replicative senescence is generally viewed as problematic, as it is not certain whether T-cell arrested states represent true replicative senescence or alternative immunosurveillance mechanisms. For instance, although CD8^+^CD27^−^CD28^−^CD57^+^ and KLRG-1^+^ T cells show some similarities to replicative senescence, they can still be stimulated to proliferate. For further reading, Zhao *et al*. provides an in-depth overview on senescent and exhausted T-cell phenotypes (Refs 84, 85).

#### The ‘hallmarks of T-cell ageing’ scheme

The various explanations of T-cell ageing based on biochemical events leading to the accumulation of molecular/cellular damage give different weight to causes of ageing, as each of them focuses on a particular aspect of the process. In order to integrate the different relationships, a novel step on the search of the causes of T-cell ageing has been made using on a multifactorial approach. Borrowing the concept of the ‘hallmarks of ageing’, a synthetic paper has proposed a list of 10 hallmarks of T-cell ageing, including primary hallmarks (thymic involution, mitochondrial dysfunction, genetic and epigenetic alterations, and loss of proteostasis) and secondary hallmarks (reduction of the TCR repertoire, naive-memory imbalance, T-cell senescence and lack of effector plasticity), which together would explain immunosenescence hallmarks (immunodeficiency and inflammageing) (Ref. 60). Similarly in the case of the hallmarks of ageing, future work in the field of T-cell ageing involves a closer examination of the interactions between the hallmarks.

### Enhancing the biochemical picture: biophysical principles of T-cell ageing

Emerging mechanobiological data suggest that the links between the ultimate causes of T-cell ageing are more complex than previously accepted in the hallmarks scheme, as biophysical properties and mechanical loading, in turn, also alter cellular states and functions, for instance shown for fibroblasts (Ref. 86), RBCs (Ref. 87) and T-cells (Ref. 26) (see Table 1). These findings imply novel feedback loops between biophysical and biochemical damage triggering T-cell ageing.

Indeed, a biophysical point of departure for investigating the mechanical age-related changes in T cells is that changes in the mechanical properties of cells are hallmarks of ageing (Refs 88, 89). Cell mechanical behaviour has been largely overlooked in the context of the immune system (Refs 90, 91). Outside of the immune system, studies have demonstrated that there is a strong correlation between age and cell stiffness, in numerous diseases, including vascular degeneration, cardiac dysfunction and cancer (Ref. 90). Studies that have applied atomic force microscopy to adherent human cells (epithelial cells (Ref. 92), fibroblasts (Ref. 93) and cardiac myocytes (Ref. 94)) seeded on flat substrates have shown that cells consistently respond to mechanical deformation with a stiffening response as a function of increasing age. Moreover, this stiffening has been observed in all cell regions (the cell edge, cytoplasm and perinuclear region) (Ref. 92). Even suspended samples of RBCs derived from healthy donors experience reduced deformability as a result of stiffening with increasing age (Ref. 95). It has been hypothesised that in some cases cell mechanical properties are altered with increased lifespan as a result of age-dependent changes to the composition and organisation of the extra-cellular matrix (Refs 96, 97).

There are many biophysical questions still open in the context of T-cell ageing, that have been only recently addressed: how does age alter the biophysical properties of immune T cells? Do mechanical properties of T-cell subsets differ, and are these differences functionally important? Do distinct membrane, nuclear and cytoskeletal structures that mediate different types of functions (synapses, migration, etc.) change with age, and if so, how? Could there be validated biophysical immune biomarkers of T-cell ageing? If so, could T-cells be mechanically reprogrammed, as recent examples in fibroblasts? (Refs 86, 98) Considering the longitudinal studies of Table 1 integrating mechanical, morphological, biomolecular *and* functional data (i.e. spontaneous migratory behaviour and immune synapses), two models of T-cell ageing are outlined next.

#### Ageing and T-cell stiffening of the plasma membrane

**Fig. 3.**
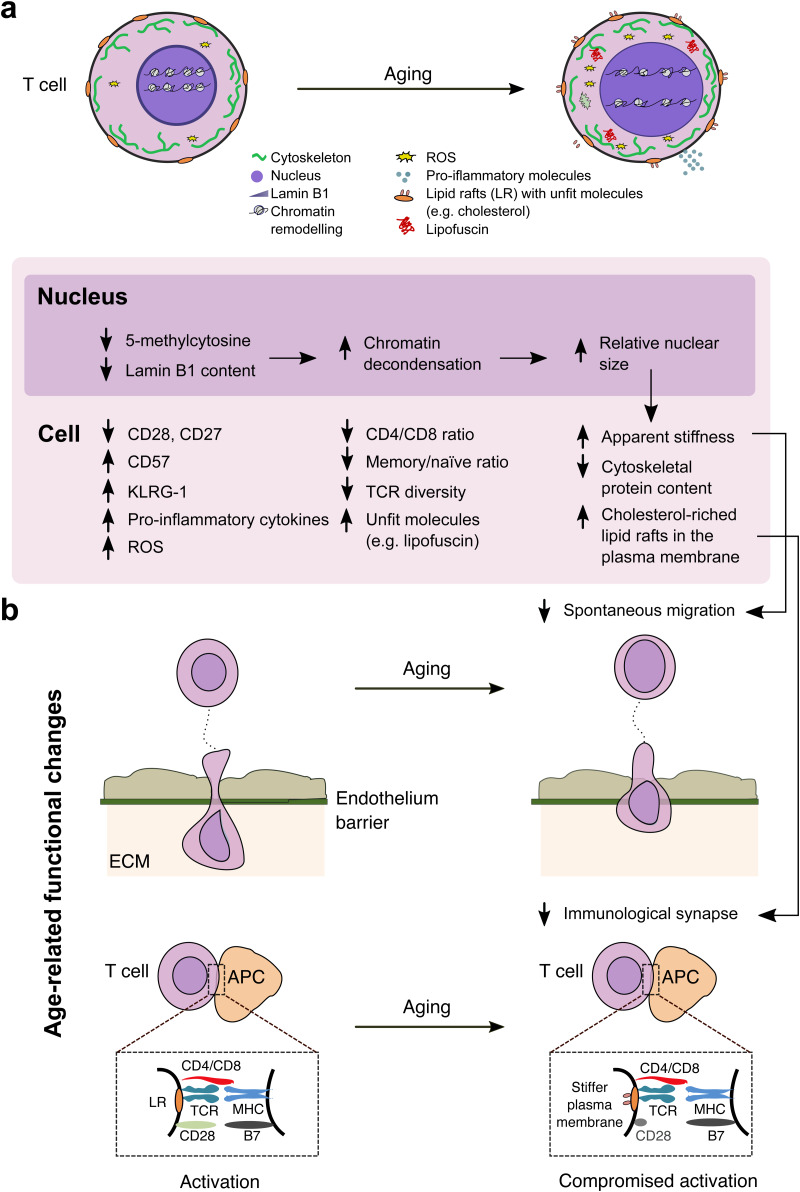
(a) Hypothetical model that integrates age-related changes in T cells. Top: Schematic representations of T-cell cytoskeleton, nucleus, lamin B1, chromatin, reactive oxygen species (ROS), pro-inflammatory molecules, lipid rafts and unfit molecules (in particular lipofuscin). Bottom: Overview of how the different components of the T-cell are affected by ageing. (b) Functional consequences of age-related changes in T cells. Top: Trans-endothelial migration declines during ageing. Bottom: T-cell activation is compromised during ageing; schematic representation of the immunological synapse between a T-cell (expressing CD4 or CD8, T-cell receptor and CD28) and an antigen-presenting cell or APC (with a major-histocompatibility complex (MHC) and membrane protein B7). The stiffer plasma membrane and loss of CD28 can compromise the activation with advancing age. The interrelations between such changes and deteriorative immune functions are mentioned (see text for description and references).

It is considered that the mechanical properties of T-cell membrane could impact immune synapses. Therefore, the use of the measure of the bending stiffness of the T-cell membrane could be useful as a marker of a decline in immune synapse during ageing, and stem from changes in its lipid composition and the distribution of lipid rafts (Refs 89, 99). The change in the composition of the plasma membrane led researchers to hypothesise that the increase of the bending stiffness could be associated with an intrinsic alteration of cholesterol metabolism in older adults, which ultimately alters the efficiency of the immune synapse (see Fig. 3) (Refs 89, 99). This description is rooted on the fact that the plasma membrane is composed of privileged signal transduction microdomains, known as lipid rafts, that participate in the creation of functional dynamic environment for immune synapses and assemble of signalling pathways. The idea provides a working framework to address the molecular mechanisms of immunosenescence. The regulation of the mechanical properties of the membrane, for example targeting cholesterol, could serve to reverse the dysfunctional immune synapses observed in the elders. However, the plasma membrane (mean thickness: 5 nm) is likely a minor contributor to the apparent stiffening of cells with age, and studies on T cells have shown that the effect of nuclear relative size (mean nuclear radius: 3 μm) is determinant compared with the effect of cytoskeleton (mean thickness: 0.3 μm) (Refs 16, 26). For further reading, the role of cholesterol in lipid rafts in T-cell ageing is reviewed in Fulop *et al*. (Ref. 99).

#### Ageing and T-cell stiffening

A recent longitudinal study included the measurement of a set of 111 parameters in four T-cell populations (CD4^+^ and CD8^+^ T cells, in both memory and naive state), that was enlarged for the case of CD4^+^ memory T cells with geometrical and internal-ordering characterisation (Ref. 26). In view of the data, a biophysical model was proposed to describe the age-related changes that ultimately lead to a loss of the ability of T cells to spontaneously migrate, as synthesised in Figure 3.

Firstly, the average relative size of the T-cell nucleus increases with age. The progressive reduction of average DNA methylation (i.e. reduction of 5-methylcytosine) may plausibly induce this relative nuclear-size increase, as previous works have reported that a larger extent of DNA methylation leads to a more compact nucleosome structure (Ref. 100). Complementarily, a reduction of nucleoskeletal lamin B1 thickness may also induce it, since this structure is crucial for maintaining nuclear morphology, as demonstrated by previous works examining how the depletion of lamin B1 resulted in larger chromatin volume (Ref. 101). The weak decrease of cytoskeletal proteins content could be associated both with an increase in the relative nuclear volume (and the consequent relative reduction of cytoplasmic volume), and with the progressive accumulation of fluorescent material, which was assumed to essentially consist of lipofuscin, as detected by self-fluorescence. Considering that the nuclear material is significantly stiffer than the cytoplasm (Ref. 102), the authors reasoned that the natural key mechanobiological mechanism that explains T-cell stiffening with age is the increase of the relative nuclear size. Indeed, in a previous multiple-measurement study of the same single T cells, it was also found that the relative nuclear size was the main contributor to T-cell apparent deformability (Ref. 16), whereas the average cytoskeletal-protein content had minor influence (Ref. 16).

According to previous studies, impaired immunity in aged individuals can be partially attributed to a relative decline in T-cell migration (Ref. 103). Overall, three ageing-related changes may plausibly play an important role in the reduction of cell migration observed in the longitudinal study: the growth of relative size of the nucleus, and the associated stiffness increase, and the reduction of myosin content. Although cell passive deformability was measured in the study, the active deformation of the cell is associated with passive deformation. Notably, the nuclear size and nucleus stiffness are highly relevant in T-cell migration, because of its higher rigidity (Ref. 104). Myosin participates in the migration process through actomyosin contraction at the rear of the T cell (Ref. 105). Furthermore, it has been shown that T lymphocytes require histone methylation in key positions in histones' proteins, a process induced by actomyosin contractility in three-dimensional environments, in order to undergo nuclear softening and confined migration (Ref. 106). In line with this view, that mechanobiological study of T-cells highlights the relationship between cell migration, nuclear stiffness and the cytoskeleton. Based on the combination of results of this study and a previous one with T cells (Ref. 16), the authors considered reasonable to assume that the increase of the relative size of the nucleus is the main contributor to T-cell stiffening with increasing age.

## Future perspectives

Because of the limited scope of empirical evidence interrelating biophysical and biochemical features of T cells during ageing, there is no single coherent model which grasps all aspects of the T-cell ageing process. Such a model could help to understand the multiple causal connections between ageing and biophysical–chemical features in T cells, and potentially control them, for instance by selecting T-cell biomarkers that indicate the benefit to the patient from a treatment, compared with their condition at baseline (e.g. predictive T-cell biomarkers of ageing rates, lifespan, all-cause mortality). Based on the information described in the previous sections, it would be reasonable to continue exploring the possible use of T-cell deformability parameters as biomarkers of biological ageing, since they reflect aggregately the molecular content and internal ordering. Future works could explore the convenience of using mechanical models allowing characterising T-cell deformability by a set of parameters, including time dependency (see Section ‘Measurement of biophysical parameters’). The use of one single mechanical parameter has been found sufficient to show a progressive stiffening of T cells during ageing (Ref. 26). However, the complexity and variety of the cellular changes (Ref. 26) suggest that improving the mechanical characterisation with a higher number of parameters could be beneficial to develop a computation of useful mechanical biomarkers.

Future studies could analyse whether different candidate alternatives of senescent T cells and exhaust T cells have a distinct signature in the biophysical and biomolecular parameters, in order to address the mechanisms involved in the relationship between cell internal ordering, phenotype and function, as well as to design interventions to rejuvenate cells (Refs 71, 72). In particular, it would be interesting to determine whether telomere shortening is associated with a change of certain features, in parallel with the relative size of the nucleus of senescent and exhaust T cells. Besides, considering the ageing of the population in the next decades, on the basis of the understanding described above, such studies would be of interest to determine which intervention might be most effective to improve immunity, in the process of immunosenescence (Ref. 107) and to define effective vaccines against those pathogens contributing to increased morbidity and mortality in the elderly (Ref. 108). Finally, large open questions that remain are whether there could be validated biophysical immune biomarkers of ageing, and if so, in which ways may cells be mechanically reprogrammed, as pioneering examples of fibroblasts have recently suggested.
